# The expression profile analysis of atrial mRNA in rats with atrial fibrillation: the role of IGF1 in atrial fibrosis

**DOI:** 10.1186/s12872-019-1013-7

**Published:** 2019-02-15

**Authors:** Jiangrong Wang, Zhan Li, Juanjuan Du, Jianhua Li, Yong Zhang, Jing Liu, Yinglong Hou

**Affiliations:** 1 Department of Cardiology, Shandong Provincial Qianfoshan Hospital, Shandong University, No. 16766 Jingshi Road, 250014 Jinan, People’s Republic of China; 2 Medical Research Center, Shandong Provincial Qianfoshan Hospital, Shandong University, Jinan, People’s Republic of China

**Keywords:** Atrial fibrillation, Atrial remodeling, mRNAs expression profile, Atrial fibrosis, IGF1

## Abstract

**Background:**

Structural remodeling is critical to the initiation and maintenance of atrial fibrillation (AF). IGF1, insulin like growth factor 1, has been recognized as contributor to fibrosis. However, the roles and mechanisms of IGF1 in structural remodeling during AF is still unclear.

**Methods:**

We investigated the transcriptional expression profiles of left atria in AF and non-AF rat models by using microarray analysis. And quantitative real-time polymerase chain reaction (qRT-PCR) was performed to validate the accuracy. After bioinformatics analysis, IGF1 was selected to explore its effects and mechanisms on atrial fibrosis. The fibroblasts were extracted from atria of rats, and randomly divided into negative control group, mIGF1 overexpression group and mIGF1 silencing group. Then 30 healthy male Wistar rats were randomly divided into negative control group (*n* = 10), pacing group (*n* = 10), pacing + mIGF1 silencing viruses group (*n* = 10). Then the intracardiac electrophysiological examination, qRT-PCR, Western Blotting, masson staining were conducted after IGF1 interfering experiments.

**Results:**

A total of 956 differentially expressed transcripts were identified, in which 395 transcripts were down-regulated and 561 transcripts were up-regulated. Bioinformatics analysis was conducted to predict the functions and interactions of the aberrantly expressed genes. The inhibition of IGF1 function in AF model could ameliorate the inducibility of AF. The IGF1 plays a fibrotic role by activating the PI3K-Akt pathway to increase the expression of CTGF and AT1R.

**Conclusions:**

IGF1 develops vital function in regulating structural remodeling during AF, which could illustrate the mechanism of AF pathogenesis and supply potential targets for its precise treatment.

## Background

Atrial fibrillation (AF) is a disorder of atrial electrical activity, which is one of the most common rapid arrhythmias. AF has seriously harm on physical and mental health of patients [1]. Although many progress has been made in the treatment of AF, the therapeutic effect of AF is still not satisfactory, which is closely related to its unclear mechanism. Structural remodeling, electrical remodeling, autonomic nerve remodeling and energy metabolism remodeling play an important role in the occurrence and maintenance of AF. Among them, structural remodeling is a key factor for the maintenance and recurrence of AF [2]. The atrial structural remodeling is mainly manifested as significant interstitial fibrosis, and the occurrence of AF increases with the degree of atrial fibrosis [3]. Atrial fibrosis can interfere with the excitation conduction of atrium, and increase the inducibility of AF [4]. Thorough treatment of AF is the main battlefield of cardiovascular field in 21 Century: are there more in-depth mechanisms to explain the occurrence and maintenance of AF? Are there any other intervention targets to prevent and cure AF?.

The aim of this study is to explore the expression profile of mRNAs transcriptome after atrial remodeling during AF and determine the key molecular that play significant roles in atrial remodeling during AF, in order to illustrate the new molecular mechanism for atrial remodeling during AF and provide potential targets for optimizing the treatment of AF.

## Methods

### Establishment of AF animal model

Twenty healthy male Wistar rats with specific pathogen free (SPF), weighing 240–260 g, were purchased from experimental animal center of Shandong University. These rats were randomly divided into two groups, control group (*n* = 10) and AF group (n = 10). The rats were anesthetized with 7% chloral hydrate (0.5 ml/100 g, intraperitoneal injection). The electrode leads were inserted into the right atria through right jugular vein in the two groups underwent surgery. The AF group underwent continuous rapid pacing (15 Hz, 900 beats/min) for 10 days to establish rat AF models, while the control group was the same as AF group but without pacing. Cardiac electrophysiological tests were performed before and 10 days after the surgery, including atrial effective refractory period (AERP) and inducibility of AF.

### Intracardiac electrophysiological examination

After 10 days of continuous atrial tachypacing (A-TP), the measuring electrode was inserted into the right atria through right jugular vein for programmed electrical stimulation. The stimulation was performed as previously described [5].The AERP was defined as the longest S1-S2 interval that failed to produce a response. We identified that AF was induced successfully if the irregular frequent atrial rhythms sustaining > 10 s, and the irregular frequent atrial rhythms lasting < 10 s was considered as paroxysmal atrial tachycardia (PAT). Then all animals were euthanized by overdose anesthetics (10% chloral hydrate, 0.5 ml/100 g, intravenous injection). The atrial samples were stored at − 80 °C.

### Microarray analysis and verification

Total RNAs were isolated from left atria by using TRIzol reagent (Invitrogen, Carlsbad, USA). The differentially expressed profiles of mRNAs were examined through the microarray analysis (Affymetrix, Sacramento, USA). Some transcripts were selected randomly, and their expression levels were analyzed by real-time quantitative PCR (qRT-PCR) to verify the sequencing results.

### Bioinformatics analysis

Gene Ontology (GO) functional analysis and Kyoto Encyclopedia of Genes and Genomes (KEGG) pathway analysis were performed to reveal and annotate the differentially expressed transcripts. The procedures were as described by us [5]. Based on the KEGG analysis of aberrantly expressed genes, the molecules in the pathways were constructed to build a network and identify the key intersections between these pathways.

### Fibroblasts isolated

The neonatal rats were anesthetized with 7% chloral hydrate. After cervical dislocation, the hearts were removed from these neonatal rats and washed with chilled phosphate buffer solution (PBS) untill blood corpuscle disappeared. Then the atrial tissue was cut into 1mm^3^ pieces with scissors. Next 2 ml of 0.1% collagenase II (Sigma-Aldrich, St. Louis, USA) solution was added to the culture dish for digesting atrial pieces. After 5 min, the supernatant was extracted from dish and mixed with high glucose DMEM (Hyclone, Logan, USA) containing 10% fetal bovine serum (Gibco, Grand Island, USA) to terminate digestion. This operation was repeated until the tissue was no longer apparent. The mixture was centrifuged at 1000 rpm for 5 min. The supernatant was discarded and the cells were resuspended with medium. The culture flask containing cells suspension was placed in incubator with 37 °C 5% CO_2_ condition. According to differential adhesion technique, the fibroblasts were separated from myocardial cells. After 24 h, we changed the culture medium and observed the adherent cells.

### IGF1 interference experiment in vitro and cell viability detection

The fibroblasts were extracted from atria of rats and cultured, and the plasmid transfection experiments were performed using 3–4 generation fibroblasts with Lipofectamine 2000 (Invitrogen, Carlsbad, USA). The cells were randomly divided into negative control group, mIGF1 overexpression group and mIGF1 silencing group. After 48 h culture, total RNAs was isolated and the protein was extracted after 72 h. CCK8 kit was used to detect the cell viability after plasmids transfection.

### Infection of adeno-associated viruses in vivo

The 9 type adeno-associated viruses (AAV) including mIGF1 silencing shRNAs were constructed in vitro by Genechem (Genechem Co. Ltd., Shanghai, China). Then 30 healthy male Wistar rats with SPF, weighing 240–260 g, were randomly divided into negative control group (*n* = 10), implantation of pacing control device, intravenous injection with negative control AAV; pacing group (A-TP, *n* = 10), continuous right atrial tachypacing (15 Hz), intravenous injection with negative control AAV; pacing + mIGF1 silencing viruses group (A-TP + mIGF1 inhibitor, *n* = 10), continuous right atrial tachypacing (15 Hz), intravenous injection with mIGF1 silencing AAV. The implantation of small animal pacemaker is the same as Part Two. A total of 1 × 10E11TU AAV were injected through caudal vein after operation. The pacing was stopped after 10 days. The animals were euthanized 14 days after the viruses injected by overdose anesthetics. Before operation and before euthanasia of animals, the electrophysiology was detected by programmed electrical stimulation in negative control group, pacing group, pacing + mIGF1 silencing viruses group, in order to detect the AERP and AF inducibility.

### qRT-PCR

The cDNA was reverse-transcribed using First Strand cDNA Synthesis Kit (Tiangen, Beijing, China) from 1 μg of total RNA. The SuperReal PreMix (SYBR Green) (Tiangen, Beijing, China) was used for the qRT-PCR analysis. And the qRT-PCR experiments were performed on the ABI ViiA 7 Real-Time PCR system (Applied Biosystems, Foster City, CA). The Table 1 displayed the specific primers for transcripts amplification. Each sample was tested in triplicate. Based on GAPDH as standardization, the relative expression levels of transcripts were calculated by 2^-ΔΔCt^ means.

**Table 1:** Primer sequence

Primer	Forward (5′-3′)	Reverse (5′-3′)
GAPDH	TGCTGAGTATGTCGTGGAGTC	GGAGATGATGACCCTTTTGG
MMP19	CTTCGAGTGGCAAAGGGCTA	GCACTAGGTCAAGTGCCCAT
COL12A1	GACACCCCCTTCTGACAGTG	GAGGTCATGCAAGACTGCCT
CALCRL	GCGCATCCTATACGGTGTCA	AAGTGTTCAGTGGGGCAGTC
SCN5A	CTGACTATAGCCGCAGCGAA	AGCTTCCACACAGAGACACG
LRRC30	CTATGTCCCGTGTGCCAAGT	TCCCCAAAACCCAAAGGGAC
NAV2	CCGAAGGAGACCCACTGATG	GCTACTCTGCTCTGGCTTCAA
IGF1	TTCACATCTCTTCTACCTG	TAGCCTGTGGGCTTGTTG
CTGF	GCGCCTGTTCTAAGACCTGT	GGCTTGGCAATTTTAGGCGT
AT1R	TGGCTGGCATTTTGTCTGG	CCTTGGGGCAGTCATCTTG
COL1A1	GAAGTCATAGGAGTCGAGGGA	CATAGGACATCTGGGAAGCAA
COL3A1	GGTTTCTTCTCACCCTGCTTC	ACAGAGGACAGATCCCGAGTC

### Western blotting

Total proteins were extracted from the left atrium and cultured fibroblasts using RIPA Lysis Buffer containing 1 mM phenylmethanesulfonyl fluoride (Beyotime Biotechnology, Beijing, China) for immunoblotting analysis. The protein content was determined by BCA Protein Assay Kit (Beyotime Biotechnology, Beijing, China). Protein sample (~ 50 μg) was fractionated by 12% polyacrylamide gels (Beyotime Biotechnology, Beijing, China) and transferred to PVDF membrane (Millipore, Bedford, USA). The membranes were blocked with 5% (*w*/*v*) fat-free milk in Tris-buffered saline (TBS-T, pH 7.4, with 0.05% [*v*/v] Tween 20) buffer for 2 h at room temperature. The membranes were incubated with the unique primary antibody in optimum dilution ratio overnight at 4 °C. Next day, the membranes were incubated with secondary antibody (HRP-labeled, Beyotime Biotechnology, Beijing, China; dilution, 1:10000) diluted in TBS-T for 1 h at 37 °C. Subsequently, the membranes were washed three times with TBS-T at room temperature for 15 min each time. The reactive proteins were visualized using enhanced chemiluminescence reagent (Millipore, Bedford, USA). Then we quantified the intensity of grayscale of per band by ImageJ software (NIH Image) and using GAPDH as standardization. Compared the experimental data with control group for normalizing, the final outcomes were displayed as fold change.

### Masson staining

The sections were obtained from left atria. The structural remodeling related Masson staining was performed. Each slide was examined under a microscope (Olympus, Tokyo, Japan) with a 20 × objective, and the 3 fields were selected to calculate the mean density.

### Cell viability

The cell viability after plasmids transfection was detected using Cell Counting Kit 8 (CCK 8) (Dojindo, Kumamoto, Japan). The fibroblasts were planked into the 96-well plate, 5000 cells per well, and with 5 wells repeated per group. After cells were transfected with mIGF1 inhibitor plasmids and mIGF1 overexpression plasmids, 10 μl CCK8 dilution was added into per well. Then the absorbance was tested under ultraviolet spectrophotometer (NanoDrop, Thermo, USA).

### Statistical analysis

Statistical analysis was performed using SPSS 19.0 software (IBM Corp., Armonk, NY). All data were presented as mean ± standard error of mean. The comparisons between two groups were conducted with unpaired *t*-test. The variance among multiple groups were performed using one-way analysis of variance (ANOVA) followed by Bonferroni’s post hoc test. *p* < 0.05 was considered as statistically significant and *p* < 0.01 as highly significant differences.

## Results

**Fig. 1:**
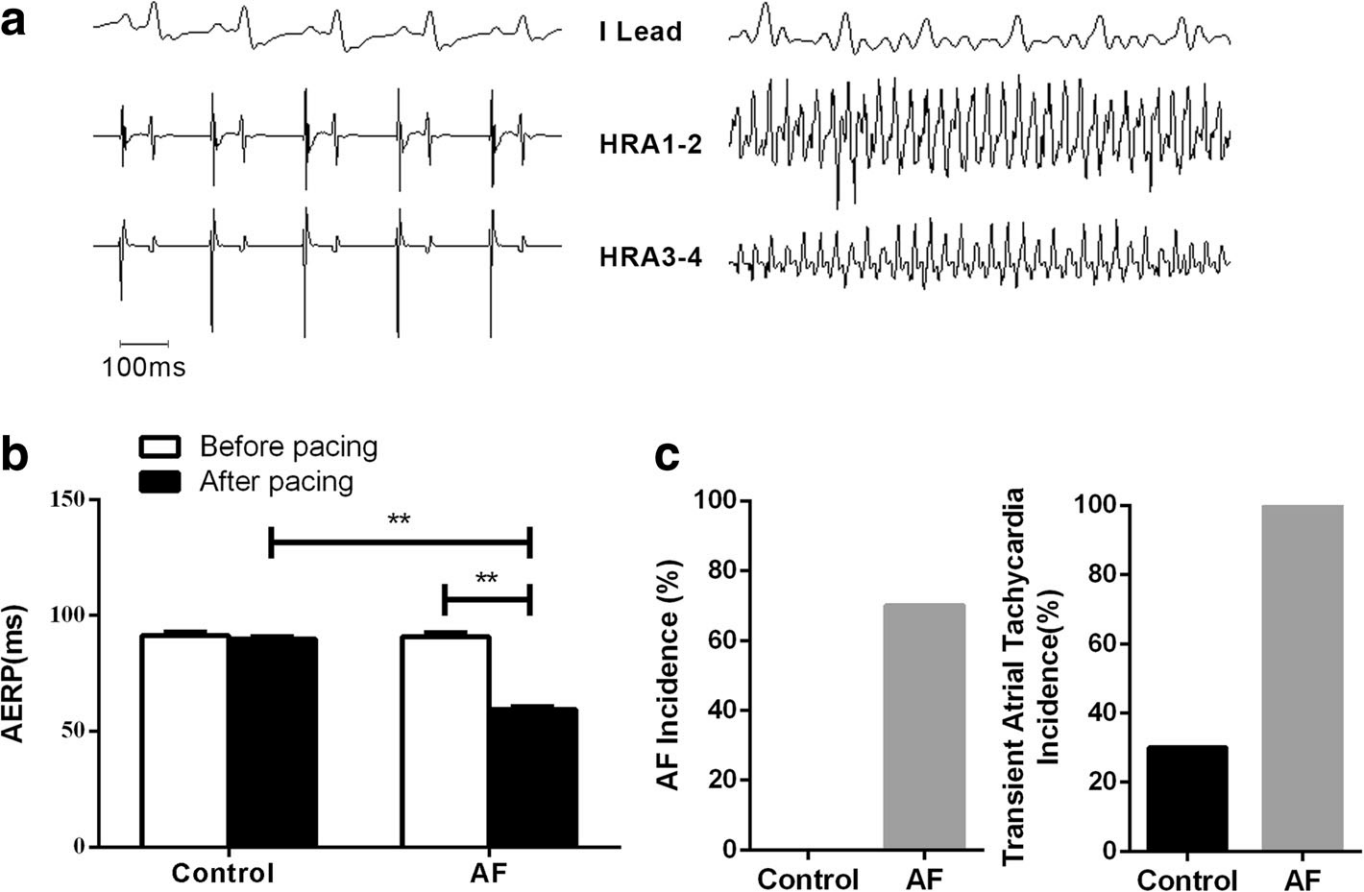
The changes of electrophysiology in AF model. (**a**) The electrocardiogramof sinus rhythm and AF rhythm; (**b**) The changes of AERP in control and AF group; (**c**)The AF inducibility of control and AF group. (*p < 0.05, **p < 0.01)

The rats AF models were successfully built through right A-TP (Fig. 1a). Compared with control group, the AERP was significantly shortened (59.50 ± 1.17 vs. 90.00 ± 1.05, *p* < 0.01) in AF group (Fig. 1b). AF was induced in 7 rats, and PAT occurred in all 10 rats in AF group, while PAT existed in 3 rats in control group (Fig. 1c).

**Fig. 2:**
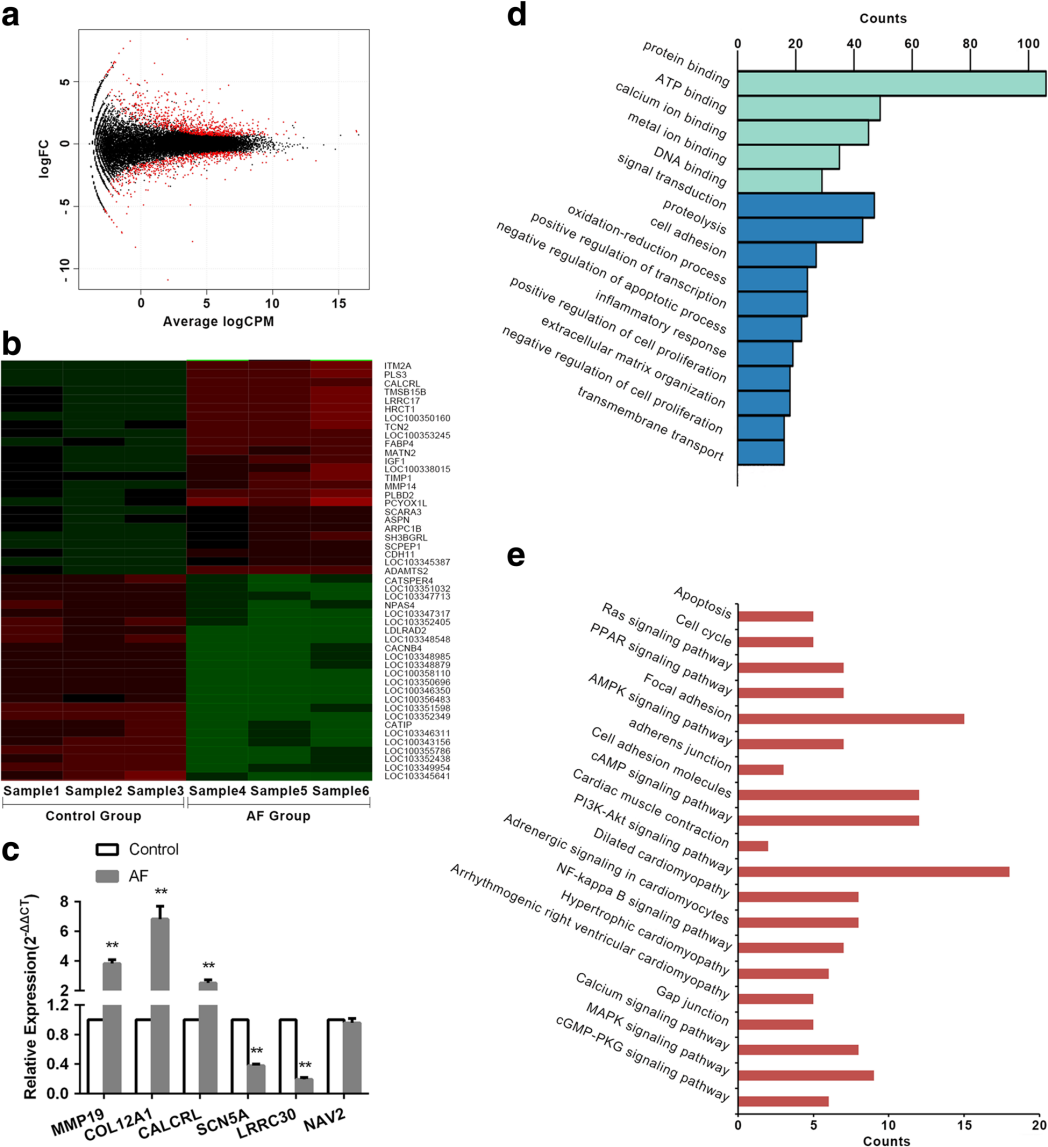
The general situation of differently expressed transcripts. (**a**) The volcano plot of differently expressed transcripts, the red dots indicate differently expressed transcripts; (**b**) The heat map of differently expressed transcripts, the red indicates high expression, and the green indicates low expression; (**c**) The verification of random selected transcripts by qRT-PCR; (**d**) The GO analysis of differently expressed transcripts; (**e**) The KEGG analysis of differently expressed transcripts. FC:Fold Change; CPM:counts per 1 million. (*p < 0.05, **p < 0.01)

Through the microarray analysis of left atria from AF and non-AF rats, 28,514 mRNAs transcripts were acquired, in which 956 transcripts meet the pipelines that Fold Change is more than 2 times and *p* < 0.05. And among them, 395 transcripts were down-regulated and 561 transcripts were up-regulated. The variance in differentially expressed transcripts was elucidated by a volcano plot based on fold change and *p*-value (Fig. 2a). The transcripts were further displayed by heat map (Fig. 2b). And the fold changes of some significant differentially expressed transcripts are listed in Table 2.

**Table 2:** The fold change of differently expressed transcripts (excerpt)

Gene name	logFC	*p* value	FDR	Gene name	logFC	*p* value	FDR
KCNJ2	1.78777632	4.19E-17	5.98E-13	CACNB4	−2.28574713	9.55E-05	0.005389546
KCNJ8	1.141819122	6.29E-06	0.00065718	CACNA1A	−1.628926883	2.97E-10	2.12E-07
KCNK6	1.150441304	0.000793349	0.023489898	KCNQ1	−1.205412872	3.18E-08	1.15E-05
CALCRL	1.241481269	2.18E-07	4.82E-05	CAMK2A	−1.412612407	3.79E-08	1.26E-05
CALM2	1.708119512	1.09E-15	1.04E-11	CAMK2N1	−1.541660634	6.52E-07	0.000115456
GJB5	1.757556094	3.25E-06	0.000411351	GJA9	−2.057285062	4.27E-07	8.22E-05
MMP9	3.73848035	4.53E-09	2.31E-06	GJA5	−2.037780185	8.80E-12	1.14E-08
MMP12	5.577927528	7.38E-08	2.12E-05	MMP21	−1.371156845	0.000904759	0.025797381
MMP14	1.489464796	7.35E-07	0.000127411	PGC-1α	−1.595394336	1.20E-11	1.43E-08
MMP17	2.291418512	1.53E-05	0.00135922	NDUFB2	−1.040414053	6.88E-05	0.004234673
MMP19	1.800160824	1.17E-08	5.04E-06	PCK1	−2.787425922	2.20E-05	0.001795008
TIMP1	1.228682606	1.78E-05	0.00153155	PLA2G4B	−1.323626109	9.15E-06	0.000893136
TGFβ1	1.933803476	8.51E-13	1.87E-09	PLB1	−1.258797201	0.000562103	0.019064513
CTGF	2.097679659	7.24E-12	1.05E-08	ARG1	−2.688485588	8.27E-05	0.004872797
AT1R	1.401791159	8.76E-08	2.40E-05	GPT2	−1.222118888	4.64E-05	0.003062075
IGF1	1.275313421	4.88E-05	0.00318585	SCN5A	−1.088333643	2.41E-06	0.000314629
COL11A1	4.038107348	2.53E-05	0.002000419	LRRC30	−2.490353038	5.47E-12	8.93E-09
COL9A2	1.80847988	0.000605855	0.019924723	WFIKKN2	−2.482540269	4.88E-15	2.78E-11
COL15A1	1.071792559	0.000616985	0.020197575	DNAH11	−1.546725963	7.72E-12	1.05E-08
NAGLU	1.033636714	0.000215238	0.009554848	GPR52	−2.607393716	3.20E-11	3.26E-08
NDUFA4L2	1.892181201	5.39E-06	0.000584728	COL12A1	3.267247408	9.93E-09	4.42E-06
NT5E	1.283136333	2.65E-06	0.000342455	COL3A1	1.734196263	0.002018163	0.044816099
P4HA3	2.497789398	0.000525282	0.018176426	COL5A1	1.402990358	0.001869416	0.042687287
PLA2G7	1.358228134	3.59E-06	0.000437818	COL6A1	1.16702211	0.001061007	0.028890663
RRM2	2.356293681	5.80E-05	0.003677039	COL6A3	1.483086607	0.000863696	0.024900466
UGT1–4	1.384070717	0.000714781	0.0222982	COL18A1	1.608183475	0.000679991	0.021643712
UGT1A7	1.33614193	0.000892918	0.025562007	LDHA	2.566727735	7.44E-12	1.05E-08
ALG5	1.3258361	4.12E-05	0.002808271	HK3	2.020949289	1.214228447	6.07E-05
ALOX5	1.909409638	6.89E-05	0.004234673	GUSB	1.089219527	9.66E-05	0.005433298
GGT5	1.895319254	3.92E-13	1.12E-09	GLS2	1.145146161	8.77E-05	0.005103408

In order to confirm the microarray analysis results, we randomly selected 3 down-regulated and 3 up-regulated transcripts to observe their variation tendency in AF models by qRT-PCR method. The outcomes showed that the variation tendency of randomly selected transcripts was consistent with the microarray results, in which the expression of MMP19, COL12A1, CALCRL were significantly up-regulated and SCN5A, LRRC30 were remarkably down-regulated. While compared with control group, the expression of NAV2 in AF group slightly decreased, but it did not reach the statistical difference (Fig. 2c).

**Fig. 3:**
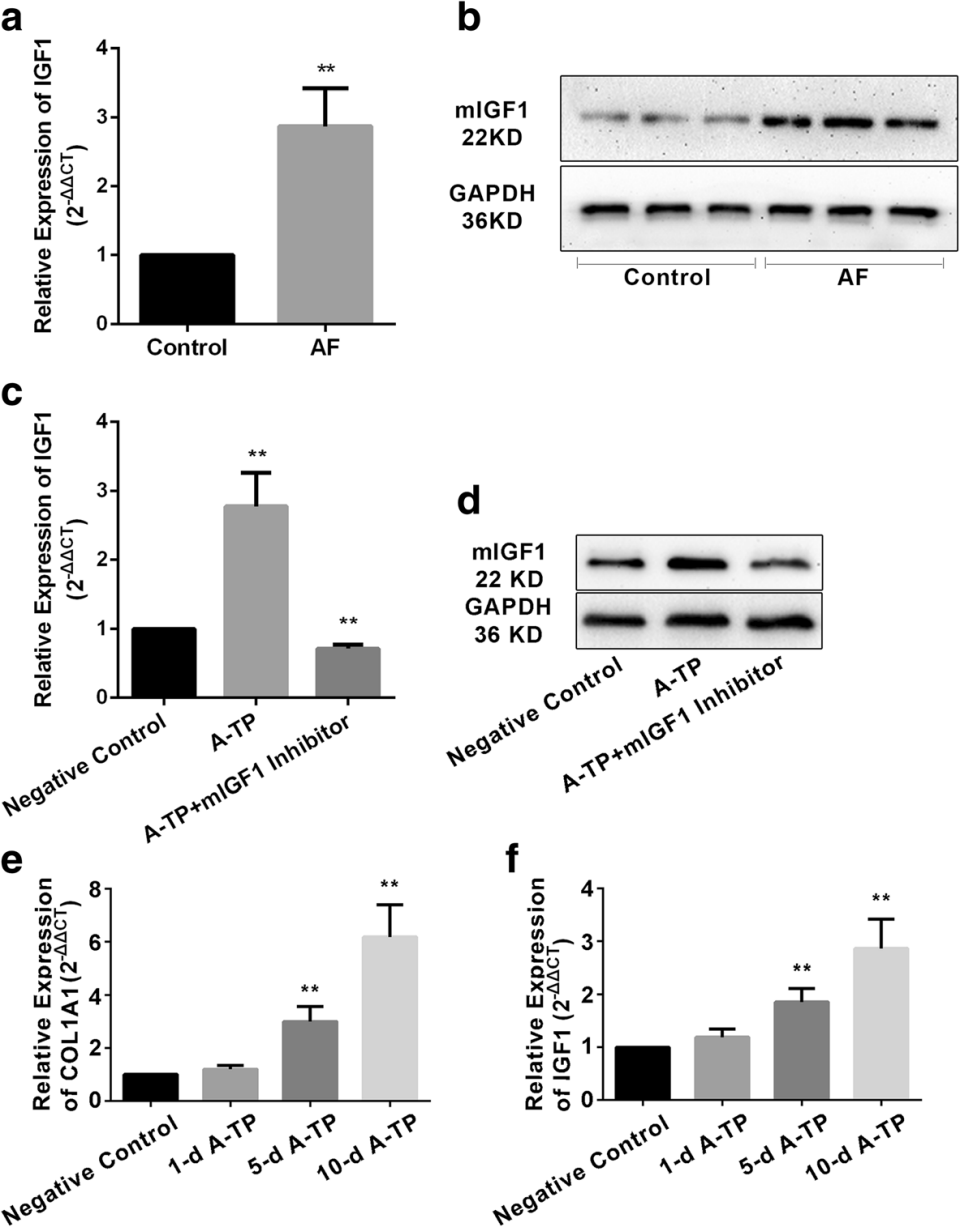
The expression of mIGF1. (**a**) The expression of mIGF1 in AF group detected by qRT-PCR; (**b**)The expression of mIGF1 in AF group detected by Western Blotting; (**c**) The expression of mIGF1 after AAV infected detected by qRT-PCR; (**d**) The expression of mIGF1 after AAV infected detected by Western Blotting. (**e**) The expression level of COL1A1 increased with the prolongation of pacing time; (**f**) The expression level mIGF1 increased with the prolongation of pacing time. A-TP: atrial tachypacing. (*p < 0.05, **p < 0.01)

GO enrichment analysis indicated that the differentially expressed genes were mainly involved in the biological processes, such as cell proliferation, apoptosis, cell adhesion, transmembrane transport, protein hydrolysis, regulation of transcription and so on (Fig. 2d). And the KEGG pathway analysis showed that the aberrantly expressed genes were associated with cell cycle, apoptosis, cell adhesion molecules, metabolic pathways, Ras signaling, PI3K-Akt signaling, NF-Kappa B signaling, PPAR signaling, arrhythmogenic right ventricular cardiomyopathy, hypertrophic cardiomyopathy, dilated cardiomyopathy (Fig. 2e). Based on the constructed pathway molecular cascade network, we screened IGF1, CACNB4, ADCY5, ITGA4 and other crossing points. These molecules may play a variety of key roles through multi-channel. We selected insulin like growth factor 1 (IGF1) as a key factor in our study to explore its effects on atrial remodeling during AF (Fig. 3a/b).

According to bioinformatics analysis, the cytokine IGF1 has the molecular function such as growth factor activity, insulin receptor binding, hormone activity, insulin-like growth factor receptor binding, integrin binding and so on. The classification and functional annotation were performed for IGF1 from the angle of GO analysis. IGF1 is involved in myotube cell development, positive regulation of myoblast proliferation, positive regulation of fibroblast proliferation, positive regulation of Ras protein signal transduction, negative regulation of smooth muscle cell apoptotic process, positive regulation of phosphatidylinositol 3-kinase cascade, positive regulation of cardiac muscle hypertrophy, myoblast differentiation, positive regulation of MAPK cascade, positive regulation of smooth muscle cell proliferation, negative regulation of extrinsic apoptotic signaling pathway, positive regulation of DNA replication and other biological processes. The IGF1 could induce fibrosis through the Ras signaling pathway, HIF-1 signaling pathway, p53 signaling pathway, mTOR signaling pathway, PI3K-Akt signaling pathway, AMPK signaling pathway, MAPK signaling pathway, focal adhesion, hypertrophic cardiomyopathy and other signaling pathways. IGF1 is mainly synthesized and secreted by liver, and skeletal and cardiac muscle could also synthesize muscle specific IGF1 (mIGF1). IGF1 plays an important role in cell proliferation, differentiation, apoptosis and metabolism by means of endocrine and paracrine form [6, 7]. The expression of transcript mIGF1 was up-regulated in left atria of AF group by qRT-PCR (Fig. 3a), and the protein level of mIGF1 was increased by Western Blotting (Fig. 3b). With the prolongation of pacing time, the expression level of alpha-1 type I collagen (COL1A1) and mIGF1 increased (Fig. 3e/f).

**Fig. 4:**
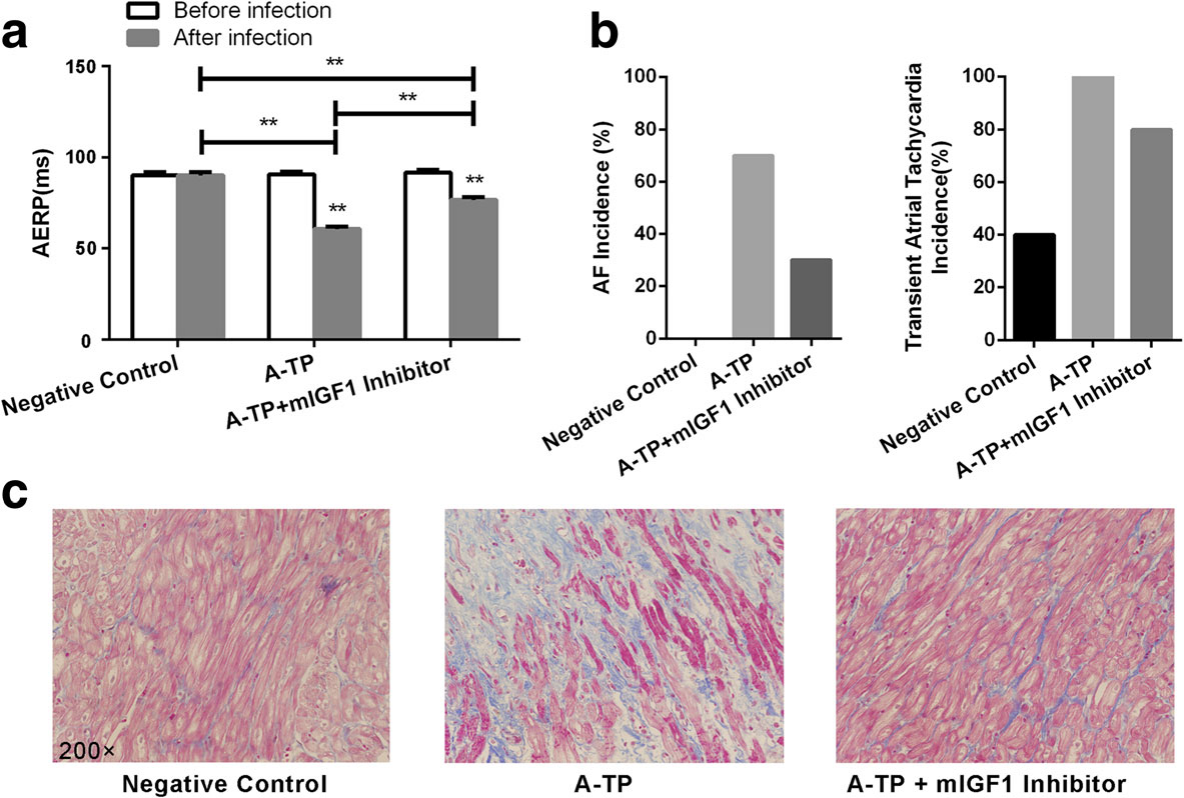
The changes of electrophysiology after AAV. (**a**) The AERP changes in negative group, pacing group, pacing + mIGF1sliencing AAV group; (**b**) The inducibility in negative group, pacing group, pacing + mIGF1sliencing AAV group; (**c**) The fibrosis degree of negative group, pacing group, pacing + mIGF1sliencing AAV group by Masson staining (200×). A-TP: atrial tachypacing. (*p < 0.05, **p < 0.01)

The AAV9 including mIGF1 silencing shRNAs were constructed in vitro. Then the in vivo infection was performed to detect the biological role of mIGF1. The qRT-PCR and Western Blotting both verified that the AAV could inhibit the expression of mIGF1 (Fig. 3c/d). Compared with negative control group, the AERP of pacing group was shortened significantly (60.50 ± 1.38 vs. 90.00 ± 1.97, *p* < 0.01) (Fig. 4a). AF was induced in 7 rats, and PAT occurred in all 10 rats in AF group, while PAT existed in 4 rats in negative control group (Fig. 4b). Compared with the pacing group, the AERP of pacing + mIGF1 silencing viruses group was prolonged (76.50 ± 1.50 vs. 60.50 ± 1.38, *p* < 0.01), but it was still lower than that of negative control group (76.50 ± 1.50 vs. 90.00 ± 1.97, *p* < 0.01) (Fig. 4a). And AF inducibility was decreased to 3 out of 10 rats, and PAT was induced in 8 rats in pacing + mIGF1 silencing viruses group (Fig. 4b).

Masson staining showed that, compared with negative control group, the degree of fibrosis in atrial tissue of pacing group was aggravated (Fig. 4c). Conversely, compared with the pacing group, the degree of fibrosis in atrial tissue of pacing + mIGF1 silencing viruses group was attenuated (Fig. 4c).

**Fig. 5:**
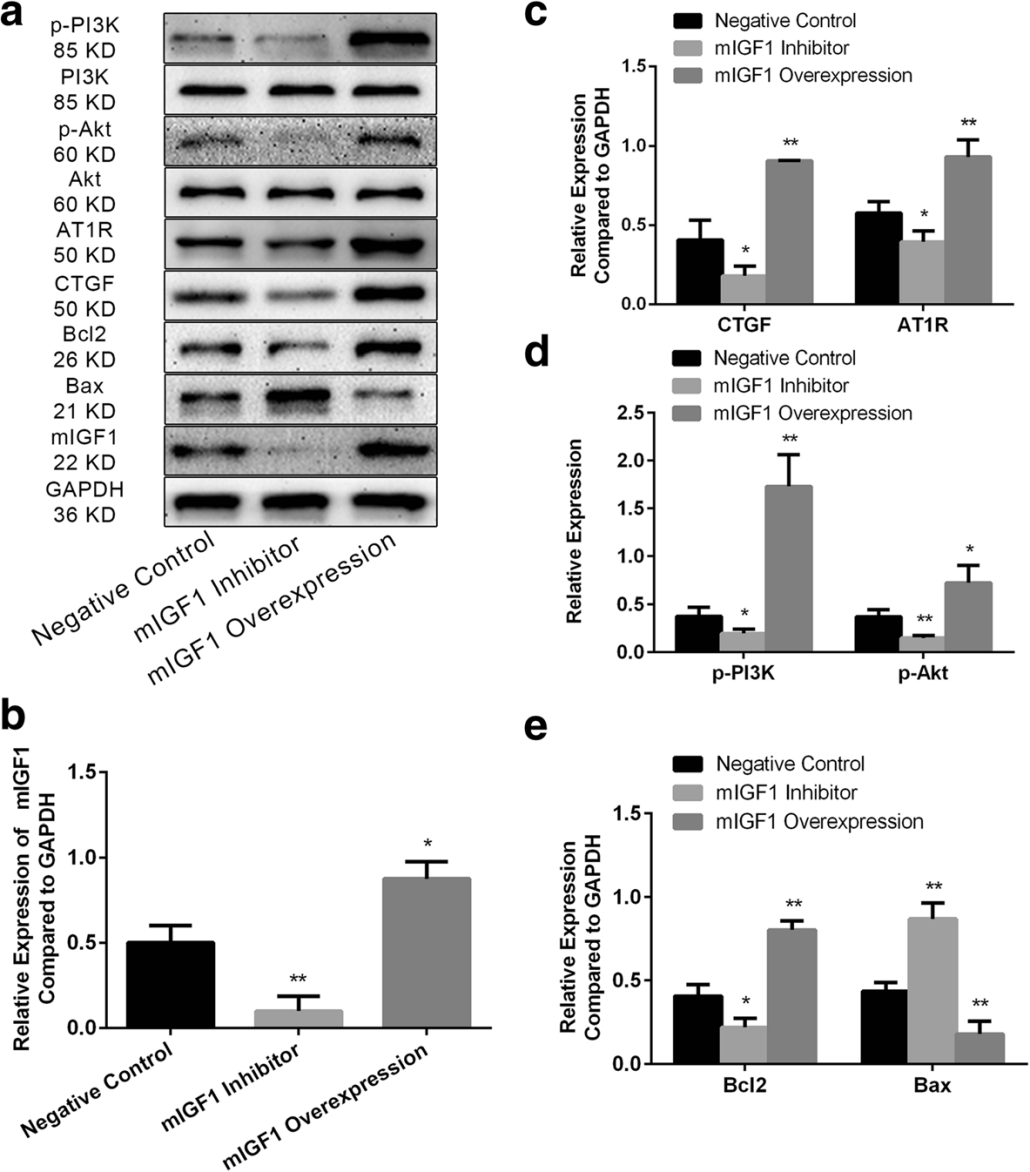
(**a**) The overview of bands after mIGF1 gene interference; (**b**) The relative expression of mIGF1 after mIGF1 gene interference; (**c**) The relative expression of CTGF and AT1R after mIGF1 gene interference; (**d**) The phosphorylation level of PI3K and Akt after mIGF1 gene interference; (**e**) The relative expression of Bcl2 and Bax after mIGF1 gene interference

**Fig. 6:**
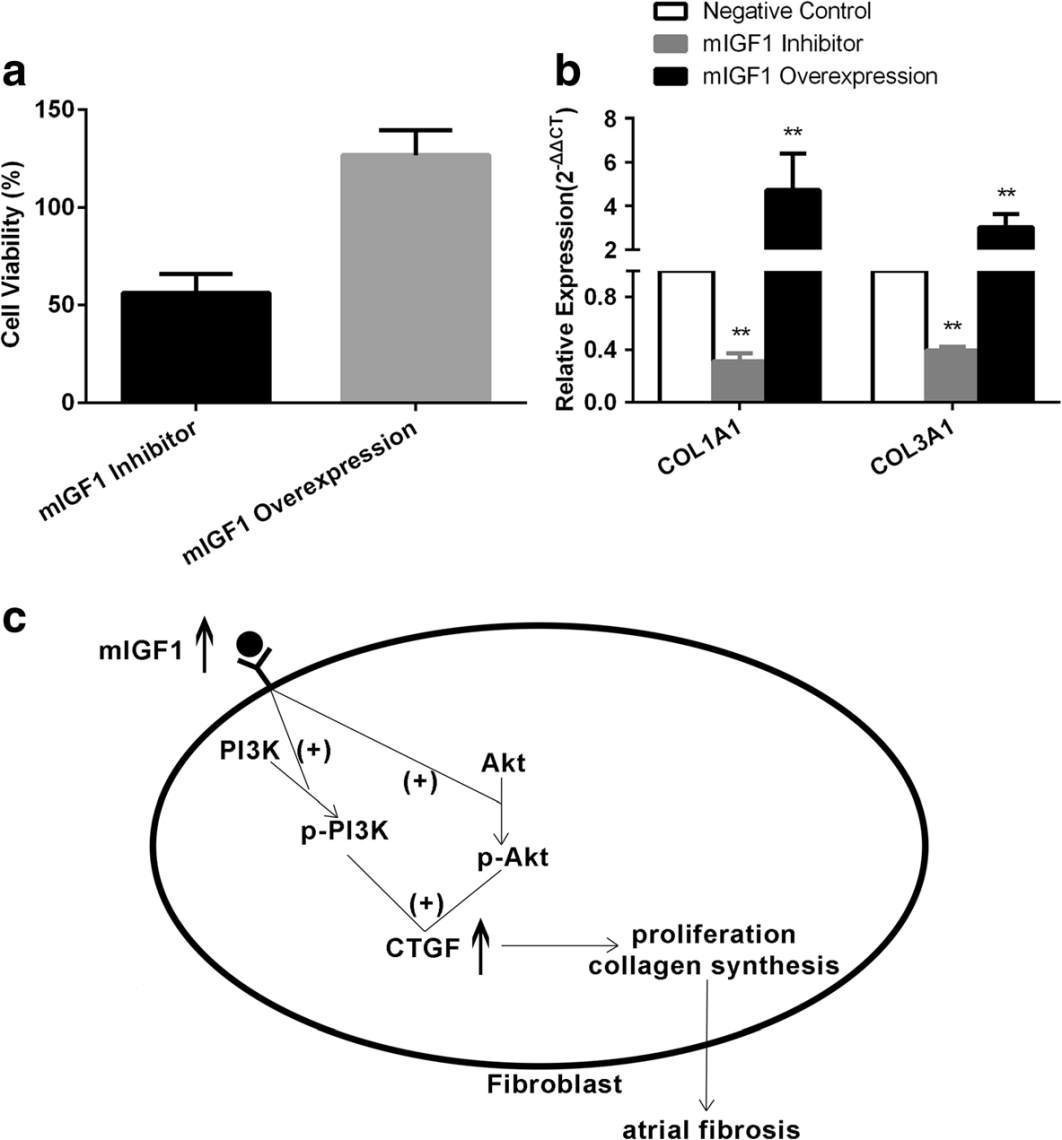
(**a**) The cell viability of fibroblasts after mIGF1 interfered detected by CCK8 Kit; (**b**) The expression of COL1A1, COL3A1 after mIGF1 interfered detected by qRT-PCR. (**c**) A schematic diagram to summarize the downstream singling pathway of IGF1 associated with atrial fibrosis during AF. (+) positive regulation. (*p < 0.05, **p < 0.01)

The mIGF1gene interference experiments were performed in fibroblasts. Compared with negative control group, after overexpression of mIGF1 (Fig. 5a/b), the expression levels of connective tissue growth factor (CTGF) and angiotensin-II type 1 receptors (AT1R) were up-regulated (Fig. 5a/c), phosphorylation levels of PI3K and Akt were up-regulated (Fig. 5a/d), the ratio of B-cell lymphoma 2 (Bcl2)/Bcl-2-associated X protein (Bax) was increased (Fig. 5a/e), cell viability was increased (Fig. 6a), and the expression levels of COL1A1 and alpha-1 type III collagen (COL3A1) were up-regulated (Fig. 6b). Conversely, after silencing mIGF1 expression (Fig. 5a/b), the expression levels of CTGF and AT1R were down-regulated (Fig. 5a/c), phosphorylation levels of PI3K and Akt were down-regulated (Fig. 5a/d), the ratio of Bcl2/Bax was decreased (Fig. 5a/e), cell viability was decreased (Fig. 6a), and the expression levels of COL1A1 and COL3A1 were down-regulated (Fig. 6b).

## Discussion

Atrial fibrillation is the most common rapid arrhythmia in clinical practice, which has attracted much attention from its high disability and mortality rate [8]. The extremely complicated pathogenesis of AF leads to poor therapeutic effect (especially chronic AF) [9]. And there is extensive evidence that fibrosis plays a central role in stabilizing the reentrant drivers to maintain the arrhythmia [10]. Atrial fibrosis can interfere with the excitation conduction of the atrium, cause one way conduction slowing or blocking, and increase the possibility of AF [11]. Studies on the molecular mechanism from nucleic acid and protein level could elucidate the basic feature of disease from the angle of genetic, growth, development, apoptosis and other biological process. A complicated network of interacting molecular signaling pathways involves in the fibrogetic process. For example, many cytokines and receptors such as CTGF, transforming growth factor-beta (TGF-β), platelet derived growth factor (PDGF) and angiotensin-II (particularly type 1 receptors) provide vital influence on fibrosis [3, 12–14]. So we further explore the expression profile of atrial transcripts in AF, and illustrate the role of IGF1 in atrial fibrosis, which is expected to provide a new explanation for the pathogenesis of AF, and provide a new strategy for the prevention of AF.

Therefore, we first explored the biological functions of mIGF1 during AF. The expression of mIGF1 in rat model of AF established by pacing was obviously up-regulated. First of all, we constructed the type 9 AAV vector in vitro, because of AAV9 is the best serotype which has high specificity targeted cardiac [15]. Then, we performed the AAV infected in pacing model, and observed the effect of mIGF1 on AF inducibility. We found that, compared with negative control group, the AERP of pacing group shortened significantly, and the AF inducibility increased; compared with the pacing group, the AERP of pacing + mIGF1 silencing viruses group prolonged, but it was still lower than that of negative control group, and the AF inducibility was improved. We observed that the inhibition of mIGF1 function in AF model could reduce the inducibility of AF.

IGF1 is a growth hormone, which promotes cell proliferation and growth, inhibits cell apoptosis and plays a role in fibrosis under various pathological conditions [16]. The high expression of IGF1 stimulates the growth of cardiac fibroblasts and promotes the synthesis of collagen fibres [17]. IGF1 plays a role of myocardial hypertrophy by activating PI3K pathway [18]. In the model of Abcb4 knockout mice (chronic biliary disease), overexpressed IGF1 up-regulated the expression of TGFβ1 and collagen 1/3/4, promoting the pathogenesis of fibrosis [19].

The cellular constituent of heart was mainly composed of myocytes, fibroblasts, and endothelial cells. Fibroblasts occupy more than 50% of the total cells [20, 21]. The cardiac fibroblasts could keep the synthesis and homeostasis of extracellular matrix and provide structural support for myocytes [20]. Extracellular matrix is a complex network of macromolecules and contains many growth factors, which not only provides suitable medium for cell survival and functional integration, but also influences cell metabolism and differentiation through signal transduction [22]. The extracellular matrix is mainly composed of collagen I and III fiber, and contains a small amount of fibronectin, laminin and so on [23]. These components are produced by fibroblasts, regulated by growth factors such as CTGF, and present different states under physiological or pathophysiological conditions [24, 25]. Under all kinds of pathological stimulation, fibroblasts overly proliferate, trans-differentiate, and synthesize redundant extracellular matrix components, such as collagen fiber, resulting in atrial fibrosis [25].

Then we further explored the potential mechanism of mIGF1 in atrial fibrosis. The mIGF1gene interference experiments were performed in fibroblasts. Compared with negative control group, after overexpression of mIGF1, the expression levels of CTGF and AT1R were up-regulated, phosphorylation levels of PI3K and Akt were up-regulated, the ratio of Bcl2/Bax and cell viability were increased, the expression levels of COL1A1 and COL3A1 were up-regulated. Conversely, after the lower expression of mIGF1, the expression levels of CTGF and AT1R were down-regulated, phosphorylation levels of PI3K and Akt were down-regulated, the ratio of Bcl2/Bax and cell viability were decreased, the expression levels of COL1A1 and COL3A1 were down-regulated. So we observed that mIGF1 can increase the expression of CTGF and AT1R by activating the PI3K-Akt pathway, thus promoting the proliferation of fibroblasts and the accumulation of collagen (Fig. 6c).

CTGF is a fibrogenic cytokine that closely related to the fibrosis process, which has obvious mitogen and chemotaxis, and could induce fibroblast proliferating and extracellular matrix depositing [26, 27]. Previous studies showed that CTGF plays an important role in the occurrence and development of hepatic fibrosis, which not only directly induces the activation, proliferation and migration of hepatic stellate cells, but also promotes the synthesis and secretion of extracellular matrix and activation of hepatic stellate cells; therefore the proportion of extracellular collagen changes in liver, and the type I and III collagen fibers especially type I collagen significantly increase [28, 29]. CTGF induces renal interstitial fibrosis and inflammatory response in mice by activating the NF-kappaB pathway [30]. The study of Wang X found that recombinant human CTGF factor stimulates H9C2 mouse cardiomyocyte line to induce tyrosine kinase cell surface receptor (TrkA) phosphorylation and exert fibrogenic effect [31].

Our study revealed the transcriptional analysis of atrial tissue during AF and the role of IGF1 in structural remodeling. For revealing the relationship between the expression level of IGF1 in human serum/atrial tissue and the occurrence or recurrence of AF, the IGF1 could be considered as a biomarker to predict the occurrence or recurrence of AF.

However, it can’t be supposed that the rat AF models after A-TP is directly analogous to clinical AF. In next, we should explore the effects of IGF1 in human cardiomyocytes and validate its role in human atria. We should further elucidate the exact way of its influence through pathway inhibitor experiments, and explore the profound mechanism of its effects on fibrosis in order to elucidate the complex mechanism of occurrence and development of AF.

## Conclusion

The mIGF1 was closely related to the structural remodeling during AF, and the role of mIGF1 in atrial fibrosis during AF was determined by functional silence experiments. The mIGF1 plays a fibrotic role by activating the PI3K-Akt pathway to increase the expression of CTGF and AT1R.

## Abbreviations

AAV: Adeno-associated viruses
ADCY5: Adenylate cyclase 5
AERP: Atrial effective refractory period
AF: Atrial fibrillation
Akt: serine/threonine kinase
AT1R: Angiotensin-II type 1 receptors
A-TP: Atrial tachypacing
Bax: Bcl-2-associated X protein
Bcl2: B-cell lymphoma 2
CACNB4: Calcium voltage-gated channel auxiliary subunit beta 4
CALCRL: Calcitonin receptor like receptor
CCK: Cell counting kit
COL12A1: alpha-1 type XII collagen
COL1A1: alpha-1 type I collagen
COL3A: alpha-1 type III collagen
CTGF: Connective tissue growth factor
GAPDH: Glyceraldehyde-3-phosphate dehydrogenase
GO: Gene Ontology
IGF1: Insulin like growth factor 1
ITGA4: Integrin subunit alpha 4
KEGG: Kyoto Encyclopedia of Genes and Genomes
LRRC30: Leucine rich repeat containing 30
mIGF1: muscle specific IGF1
MMP19: Matrix metallopeptidase 19
NAV2: Neuron navigator 2
PAT: Paroxysmal atrial tachycardia
PBS: Phosphate buffer solution
PI3K: Phosphoinositide 3-kinase
SCN5A: Sodium voltage-gated channel alpha subunit 5
SPF: Specific pathogen free
TBS-T: Tris-buffered saline + Tween 20
